# Assessment of Adverse Childhood Experiences & Resilience in Adults With Substance Use Disorders & Healthy Controls: A Case-Control Study

**DOI:** 10.1192/j.eurpsy.2026.10492

**Published:** 2026-07-31

**Authors:** S. L. Langstieh, S. Singh, M. S. Sen

**Affiliations:** Psychiatry, IHBAS, Delhi, India

## Abstract

**Introduction:**

Substance use disorders (SUDs) remain a major public health challenge globally and in India, with relapse rates remaining high despite available treatments. Adverse Childhood Experiences (ACEs) are widely recognized as risk factors for SUDs, impacting neurobiological and psychosocial development. Resilience, the capacity to adapt and recover from adversity, may buffer this risk but is underexplored in Indian clinical populations.

**Objectives:**

To compare the prevalence and severity of ACEs and levels of resilience in adults with substance use disorders and healthy controls within an Indian clinical setting.

**Methods:**

A nine-month case-control study was conducted at the Institute of Human Behaviour & Allied Sciences, Delhi. Thirty-five adults diagnosed with SUD per DSM-5 criteria and 35 matched healthy controls were recruited. Participants were assessed using the WHO ACE-International Questionnaire (ACE-IQ) for childhood adversity and Connor-Davidson Resilience Scale (CD-RISC) for resilience. Additional clinical and demographic data were collected via semi-structured interviews and validated scales including ASSIST, GHQ-12, SDS, and RCQ.

**Results:**

The SUD group reported significantly higher frequency and severity of ACEs than controls (p = 0.001), especially emotional abuse and exposure to community violence. Resilience scores did not differ significantly overall, but lower resilience correlated with greater ACE severity among SUD patients. Higher ACE exposure was associated with socio-educational deprivation and clinical risk factors, including perpetuating influences on substance use. The readiness to change was reflected in higher contemplation and action scores in the SUD group.

**Table 1.** ACE Binary, ACE Frequency & Resilience (CD-RISC) scores amongst the participants Table 1. Long description.

Score	Mean ± SD Of Cases (N=35)	Mean ± SD Of Controls (N=35)	t-test (df ) (p-value)
ACE Binary Score	5.14 ± 2.25	4.17 ± 2.41	1.744 (68) (0.086)
ACE Score Freq	3.31 ± 2.18	1.54 ± 1.63	3.848 (68) (<0.001)***
CD-RISC	73.23 ± 8.69	71.51 ± 13.13	0.644(58.99) (0.522)

*** = p < 0.001 (very highly significant)

**Table 2.** Severity of ACE Frequency Scores Amongst The Study Participants Table 2. Long description.

Group	Absent (0)	Mild (1–3)	Severe (≥4)	χ² value (df ) p-value
Controls	13 (37.1%)	16 (45.7%)	6 (17.1%)	10.80; (2); p ≈ 0.0065**
Cases	3 (8.6%)	16 (45.7%)	16 (45.7%)	

** = p < 0.01 (highly significant)

**Conclusions:**

Frequent and severe ACEs are strongly linked to SUDs in this Indian sample, with childhood trauma moderating resilience. These findings highlight the critical need for trauma-informed, resilience-enhancing interventions integrated into substance use treatment programmes. Early identification of childhood adversity and promotion of protective psychosocial resources may improve long-term outcomes.

**Disclosure of Interest:**

None Declared

## Long descriptions

### Table 1. Long description

The table consists of four columns: Score, Mean plus or minus S D of Cases (N equals 35), Mean plus or minus S D of Controls (N equals 35), and t-test (d f) (p-value).

* Row 1: A C E Binary Score. Cases: 5.14 plus or minus 2.25. Controls: 4.17 plus or minus 2.41. t-test: 1.744 (68) (0.086).

* Row 2: A C E Score Freq. Cases: 3.31 plus or minus 2.18. Controls: 1.54 plus or minus 1.63. t-test: 3.848 (68) (less than 0.001). This result is marked with three asterisks indicating it is very highly significant.

* Row 3: C D dash R I S C. Cases: 73.23 plus or minus 8.69. Controls: 71.51 plus or minus 13.13. t-test: 0.644 (58.99) (0.522).

A footnote specifies that three asterisks indicate a p-value less than 0.001.

Navigate back to Table 1..

### Table 2. Long description

The table consists of five columns and three rows of data.

Columns are labeled as follows: Group, Absent (0), Mild (1 to 3), Severe (greater than or equal to 4), and chi-squared value (d f) p-value.

Row 1 (Controls): Absent (0) is 13 (37.1 percent); Mild (1 to 3) is 16 (45.7 percent); Severe (greater than or equal to 4) is 6 (17.1 percent).

Row 2 (Cases): Absent (0) is 3 (8.6 percent); Mild (1 to 3) is 16 (45.7 percent); Severe (greater than or equal to 4) is 16 (45.7 percent).

The final column spans both rows and indicates a chi-squared value of 10.80 with 2 degrees of freedom and a p-value approximately equal to 0.0065. A footer note indicates that two asterisks signify a p-value less than 0.01, which is highly significant.

Navigate back to Table 2..

